# Associations between triglyceride-glucose indices and delirium risk in critically ill patients with acute kidney injury: a retrospective study

**DOI:** 10.3389/fendo.2025.1521850

**Published:** 2025-04-10

**Authors:** Wenhui Zhang, Tao Jin, Xinyue Hu

**Affiliations:** ^1^ School of Medicine, Anhui University of Science & Technology, Huainan, Anhui, China; ^2^ Key Laboratory of Industrial Dust Deep Reduction and Occupational Health and Safety of Anhui Higher Education Institutes, Huainan, Anhui, China; ^3^ Joint Research Center for Occupational Medicine and Health of Institute of Health and Medicine, Anhui University of Science and Technology, Huainan, Anhui, China

**Keywords:** delirium, acute kidney injury, intensive care unit, triglyceride-glucose indices, insulin resistance, Cox proportional hazard

## Abstract

**Background:**

Delirium frequently occurs in individuals with acute kidney injury (AKI), leading to serious adverse outcomes. However, there are currently no predictors of early intervention for delirium in clinical practice. This study aims to investigate whether a correlation exists between TyG indices and the clinical symptoms of delirium in patients with AKI.

**Methods:**

Eligible participants diagnosed with AKI from the Medical Information Mart for Intensive Care-IV (MIMIC-IV) database were categorised based on their TyG index. The primary outcome of this study was the incidence of delirium. The TyG indices were quartile and Kaplan–Meier (K-M) cumulative curve was conducted to compare the consequence of each group. Cox proportional hazards and restricted cubic spline (RCS) analyses were employed to explore the associations between TyG indices and outcomes. To mitigate potential biases, a no-replacement propensity score matching (PSM) approach was employed. Subgroup analyses were conducted to explore differences across various demographic and clinical categories.

**Results:**

A positive correlation between the quartile groupings of TyG-AVG and an increased cumulative incidence of delirium in individuals with severe AKI, as demonstrated through K-M cumulative curves and Cox regression analysis. Regarding the TyG index, patients in the 4th group displayed the highest hazard of delirium in both of the methods mentioned above. Furthermore, RCS analysis indicated that the interaction between the two variables is approximately linear. Subgroup analyses revealed that the effects of both metrics remained consistent across most examined subgroups.

**Conclusion:**

Higher TyG indices were clearly associated with the incidence of delirium in patients with severe AKI. These indices could serve as valuable tools for identifying delirium-prone individuals with AKI.

## 1 Introduction

Delirium is an acute neuropsychiatric syndrome (1, 2) associated with adverse outcomes in patients with acute kidney injury (AKI) (3), including increased use of mechanical ventilation, prolonged hospitalisation, higher short-term mortality rates (4), and long-term cognitive decline (5, 6). Effective prevention strategies can significantly reduce the duration and frequency of delirium episodes (7). However, currently, there is a lack of reliable early predictive biomarkers for delirium in AKI patients, limiting timely interventions and management strategies.

The triglyceride-glucose (TyG) index, a surrogate marker for insulin resistance (IR) (8, 9), has been extensively studied in various disease populations (10). Elevated TyG levels have been associated with poor prognosis in AKI patients (11) and have also been identified as a predictor for delirium in elderly patients following type 2 diabetes surgery and in sepsis patients (12, 13). However, its potential role in AKI-related delirium remains unexplored. Given the dynamic changes in IR among critically ill AKI patients, a single static TyG measurement may not fully capture its impact on delirium risk. Recent studies have introduced the triglyceride-glucose average (TyGVR) as a novel dynamic biomarker reflecting longitudinal fluctuations in IR, demonstrating superior predictive value for metabolic and cardiovascular outcomes (14, 15). To further refine this approach, we propose TyG-AVG, an innovative aggregate indicator that incorporates the mean triglyceride and glucose levels from multiple measurements, offering a more stable representation of insulin resistance compared to single-point TyG assessments. This approach may enhance the predictive accuracy for delirium risk in AKI patients.

Therefore, this study aims to explore the association between the TyG index, the aggregate biomarker TyG-AVG, and delirium in AKI patients. By elucidating these relationships, this research may contribute to a better understanding of metabolic dysregulation in critically ill AKI patients. If confirmed in future studies, these findings could potentially inform early risk stratification strategies for delirium in this population.

## 2 Materials and methods

### 2.1 Source of data

For the sake of large sample size and complete data, and for cost-effective considerations, the researchers chose to conduct a retrospective observational study using the Medical Information Mart for Intensive Care IV (MIMIC-IV version 3.0) database (16) (https://mimic.mit.edu), comprises 94,458 critically ill patients admitted between 2008 and 2022, to explore the relationship between the TyG index and the incidence of delirium in patients with AKI.

### 2.2 Cohort selection

**Figure 1:**
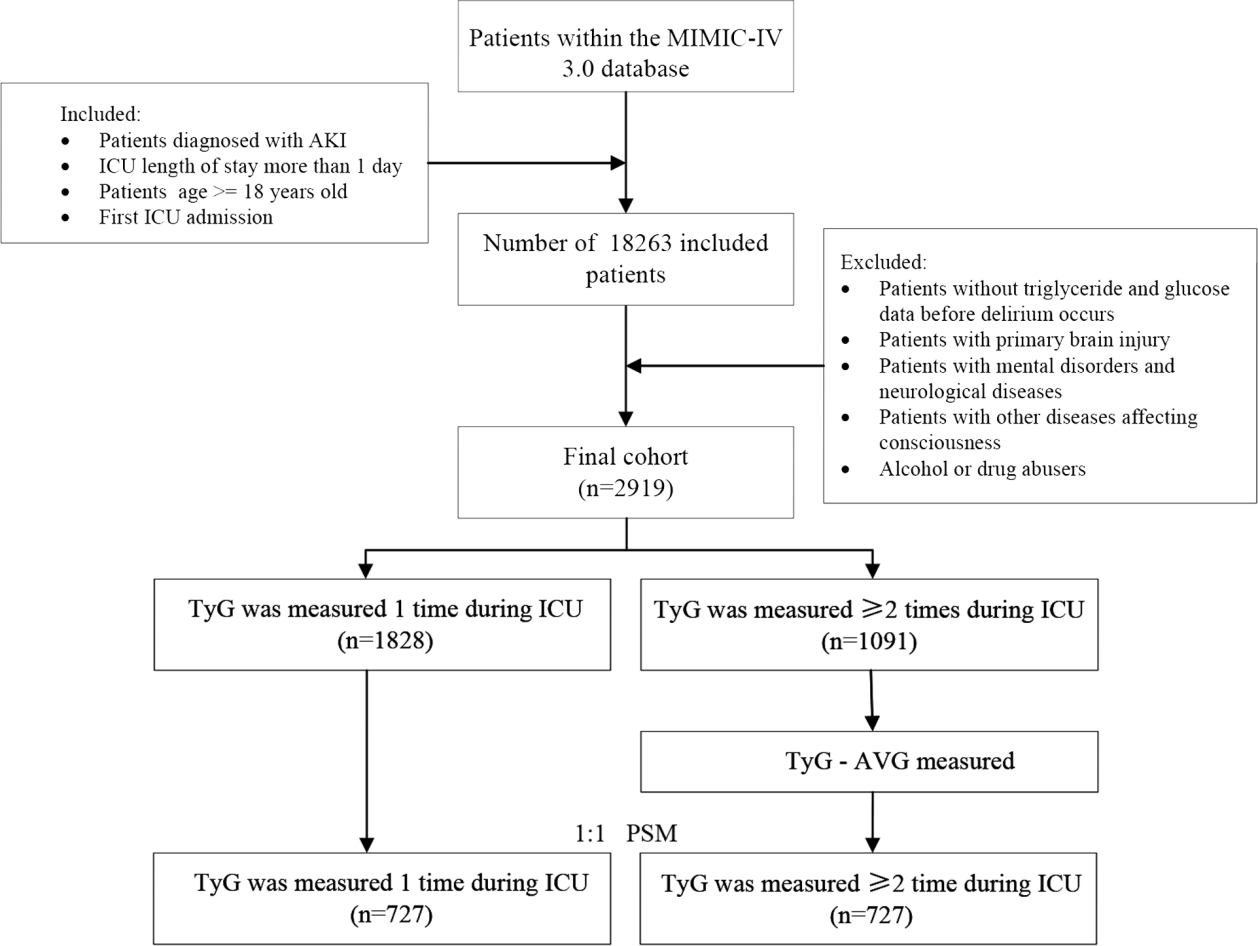
Flowchart depicting the patient screening process.

In this study, 18,263 patients with AKI who were admitted to the ICU for the first time and stayed for > 24 hours (age ≥ 18) were included. To reduce bias, patients with brain injury, mental disorders, neurological conditions, other disorders affecting consciousness, or a history of alcohol or drug abuse were excluded. Additionally, patients without glucose and triglyceride measurements from ICU admission to the onset of delirium were excluded to ensure data integrity. Overall, 1,828 eligible patients with a single glucose and triglyceride record in the ICU were assigned to one group, while 1,091 eligible patients with two or more records were assigned to another group (**Figure 1**).

### 2.3 Data collection

PostgreSQL (version 14) was used to filter individuals from the MIMIC-IV database and retrieve baseline characteristics. The extracted data included baseline variables commonly found in ICU and potential risk factors for delirium, grouped into nine primary categories: (1) demographics (age, sex, race, and so on); (2) laboratory tests (PCO2, PO2, pH, and so on); (3) vital signs (heart rate (HR), systolic blood pressure (SBP), and so on); (4) comorbidities (cancer, sepsis, peripheral vascular disease, and so on); (5) medications (propofol, insulin, and diuretics); (6) length of hospital and ICU stay; (7) severity scores (SOFA, SAPS II, GCS, SIRS, and APS III); (8) interventions (RRT, ventilation); and (9) outcomes (delirium). Furthermore, the International Classification of Diseases (ICD) 9 and 10 codes were used to extract these complications.

To address missing values for height, weight, vital signs, and laboratory tests, multiple imputation is employed to reduce the likelihood of potential bias. This approach is simple to use and maximizes the preservation of the data structure, but it also introduces bias. The primary outcome was the occurrence of delirium.

### 2.4 Definitions

In this study, baseline characteristics were assessed based on the quartiles of the TyG index. The initial recorded fasting triglyceride level was multiplied by the average fasting blood glucose value obtained within 24 hours before and after the first triglyceride measurement, and the result was divided by two. Subsequently, the natural logarithm of this calculation was computed, yielding the final TyG index.

The TyG-AVG reflects the long-term trend of the TyG index. The calculation steps were as follows: the mean triglyceride level, obtained after the first fasting measurement, was multiplied by the mean fasting blood glucose level recorded 24 hours after the first triglyceride measurement and before the onset of delirium. Subsequently, the resulting value was multiplied by 1/2. Finally, the natural logarithm of this calculation was taken.


$$\begin{array}{c} \text{TyG}-\text{AVG}=\text{ln}(\text{blood}\;\text{glucose}\;\text{average}\\ \times \text{blood}\;\text{triglyceride}\;\text{average}\times 1/2) \end{array}$$

All units were expressed in mg/dL. It is important to note that the hyphen is denoted by “-”, “ln” represents the natural logarithm with base e, “×” signifies the multiplication operation, and “/” indicates the division operation.

In this study, patients with AKI were extracted based on ICD-9 or ICD-10 codes that met the Kidney Disease: Improving Global Outcomes (KDIGO) guidelines. The Confusion Assessment Method for the ICU (CAM-ICU) was the only method available in the MIMIC-IV 3.0 database for assessing delirium.

### 2.5 Statistical analysis

The median of the interquartile range was used to express continuous variables. Grade differences between all samples were assessed using the Kruskal–Wallis test, a statistical analysis method. Frequency and percentage served as key tools for categorical variables, reflecting the distribution across different categories within the population. The chi-square test was used to conduct an in-depth analysis of the differences between groups. Furthermore, a no-replacement propensity score matching (PSM) programme was carefully designed, before being executed using Stata (version 18.0) software. The calliper width was set at 0.2. This approach aimed to ensure that the two datasets were potentially comparable.

The baseline characteristics of the initial and matched populations are presented in this study. Additionally, Kaplan–Meier (K–M) cumulative curve analysis was used to accurately assess the trend of the cumulative incidence rate of delirium. Furthermore, the results were statistically evaluated using a log-rank test. Restricted cubic spline (RCS) analysis was conducted to assess the potential nonlinearity of the TyG index and TyG-AVG regarding the incidence of delirium, with the Wald test employed to determine the presence of such nonlinearity.

To further investigate the relationships between both metrics and the risk of ICU delirium, a Cox proportional hazards analysis was conducted for both groups of patients after PSM. Model 1 was unadjusted, while Model 2 was adjusted for sex, age, and body mass index (BMI). Lastly, Model 3 included adjustments for sex, age, BMI, heart rate, respiratory rate, race, potassium levels, renal disease, myocardial infarction, congestive heart failure (CHF), SBP, diastolic blood pressure (DBP), white blood cells (WBC), red blood cells (RBC), platelets, blood urea nitrogen (BUN), partial pressure of carbon dioxide (PCO_2_), partial pressure of oxygen (PO_2_), pH, peripheral vascular disease, hypertension, cancer, Sequential Organ Failure Assessment (SOFA), Glasgow Coma Scale (GCS), Simplified Acute Physiology Score II (SAPS II), chronic pulmonary disease, liver disease, Systemic Inflammatory Response Syndrome (SIRS), Acute Physiology Score III (APS III), sepsis, renal replacement therapy (RRT), ventilation, atrial fibrillation, insulin, propofol, and diuretics.

Subgroup analysis was conducted to evaluate whether the predictive performance of both metrics remained consistent across specific groups, including age (≤ 60 years and > 68 years) and hypertension. The box plot primarily conveys essential information regarding the central tendency and variability of the data. A *P*-value threshold of 0.05 was established, with values below this indicating statistical significance for differences between groups. Data processing was conducted using R and SPSS.

## 3 Results

### 3.1 Cohort characteristics

**Supplementary Table 1** presents the characteristics of 2,919 eligible individuals, clearly illustrating the differences between groups within each TyG index quartile. Among all patients, the age quartile and BMI were 69.8 years [58.8, 79.5] and 28.1 kg/m² [24.0, 33.1], respectively. Of the total, 64.8% were male, and 41.8% experienced delirium. In comparison, patients in the other quartiles were younger, exhibited a higher BMI, and demonstrated elevated respiratory rates and body temperatures than those in the first quartile (Q1). Furthermore, patients in the higher TyG index group exhibited a lower likelihood of developing CHF and atrial fibrillation. However, they were more likely to develop diabetes and sepsis.

**Supplementary Table 2** presents the two groups divided by the frequency of TyG index measurements. After PSM, the imbalance between the TyG index and TyG-AVG groups regarding the TyG index, age, race, BMI, vital signs, laboratory tests, comorbidities, medications, severity scores, length of stay, and outcomes was significantly reduced, resulting in a comparable balance, with both the chi-square test and the Kruskal–Wallis test greater than 0.05. Additionally, the incidence of delirium among patients with AKI increased from 42.6% in the MIMIC database 2.2 to 47.2% in the MIMIC database 3.0.

### 3.2 Association of the metrics with delirium incidence

**Figure 2:**
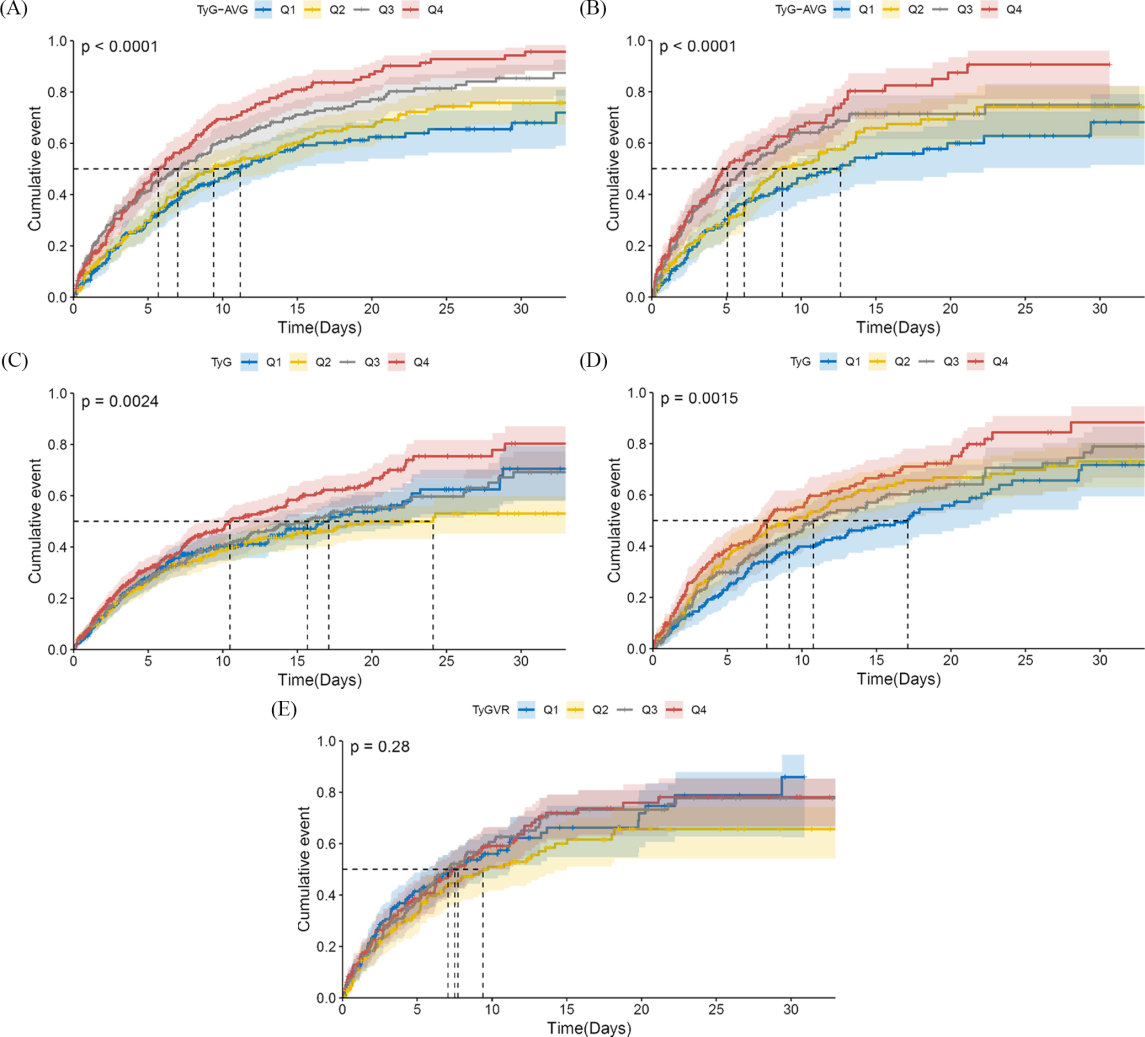
Cumulative incidence curves of delirium in the acute kidney injury population. TyG-AVG: Before PSM, Q1 (7.24–9.13), Q2 (9.13–9.68), Q3 (9.68–10.21), and Q4 (10.21–13.34); After PSM, Q1 (7.24–8.98), Q2 (8.99–9.50), Q3 (9.50–9.98), and Q4 (9.98–11.82). TyG index: Before PSM, Q1 (4.03–8.62), Q2 (8.62–9.07), Q3 (9.07–9.56), and Q4 (9.56–13.54); After PSM, Q1 (6.85–8.79), Q2 (8.79–9.31), Q3 (9.31–9.83), and Q4 (9.83–13.54). TyGVR: Q1 ((-0.23)–(-0.03)), Q2 ((-0.03)–0.01), Q3 (0.01–0.06), and Q4 (0.06–0.59). The cumulative curve for delirium is plotted based on the following indices at the quartiles of different cohorts: **(A)** TyG-AVG before PSM; **(B)** TyG-AVG after PSM; **(C)** TyG index before PSM; **(D)** TyG index after PSM; and **(E)** TyGVR after PSM.

**Figure 2** displays cumulative incidence curves categorised by quartiles of the three metrics, representing the probability distribution of delirium occurrence. In the unmatched cohort for TyG-AVG, patients with AKI exhibited a higher likelihood of developing delirium with elevated TyG-AVG values (log-rank P < 0.0001, **Figure 2A**). In the matched population, this correlation persisted, while the time point at which delirium occurred was shifted backward by 50% (log-rank P < 0.0001, **Figure 2B**). Regarding the TyG index, in the pre-match cohort, as the TyG value increased, patients with AKI were more likely to develop delirium (log-rank P = 0.0024, **Figure 2C**). Patients in the 4th group of the matched cohort exhibited the highest hazard of delirium (log-rank P = 0.0015, **Figure 2D**). However, the differences among the four groups of TyG variability ratio (TyGVR) did not achieve statistical significance in the matched population (log-rank P = 0.28, **Figure 2E**).

**Figure 3:**
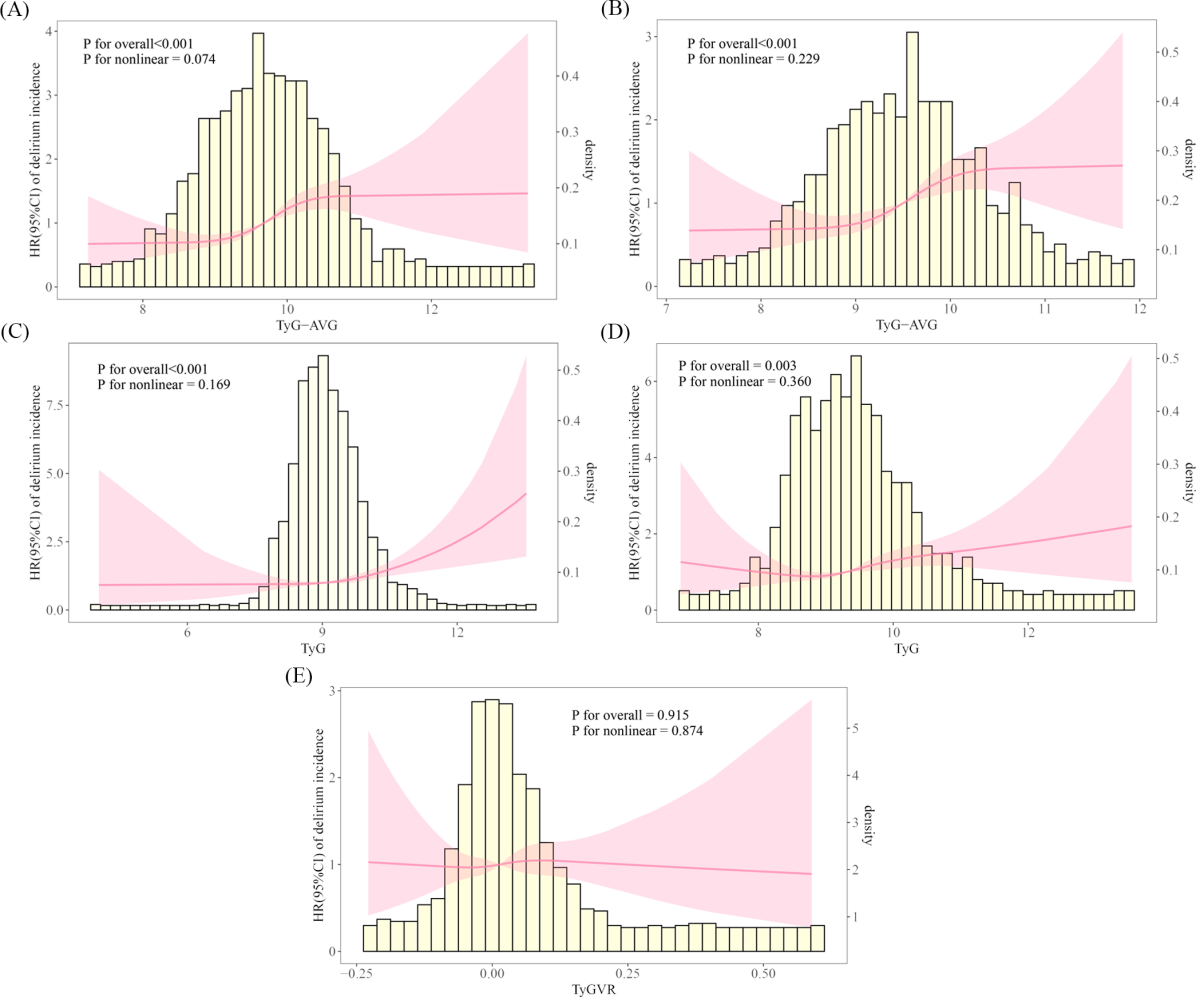
Restricted cubic spline curves illustrating the incidence of delirium in the acute kidney injury population. The RCS plot illustrates the relationship between delirium incidence in patients with AKI and the following indicators: **(A)** TyG-AVG before PSM; **(B)** TyG-AVG after PSM; **(C)** TyG index before PSM; **(D)** TyG index after PSM; and **(E)** TyGVR after PSM.

In the RCS analysis, the results for the pre-matched cohort indicated that the role of TyG-AVG in the occurrence of delirium was pronounced (overall P-value < 0.001). The interaction between the two variables was approximately linear (nonlinear P-value = 0.074) (**Figure 3A**). In the matched cohort, TyG-AVG remained significantly associated with the occurrence of delirium (overall P-value < 0.001). The linear relationship between the two variables persisted (nonlinear P-value = 0.229) (**Figure 3B**). Additionally, an analysis of the TyG indicator revealed similar conclusions both before and after PSM (**Figures 3C, D**). After PSM, while a possible linear correlation was observed (nonlinear P-value = 0.874) between TyGVR and the incidence of delirium in patients with AKI, this correlation was not statistically significant (overall P-value = 0.915) (**Figure 3E**). These findings suggest that the current evidence is insufficient to establish or refute a direct causal relationship between TyGVR and delirium.

The TyG index and TyG-AVG were identified as significant risk factors in the unadjusted Model when analysed as continuous variables using Cox proportional hazard analysis after PSM. Specifically, the hazard ratio (HR) for the TyG index was 1.24 (95% confidence interval [CI]: 1.10–1.41), while the HR for TyG-AVG was 1.41 (95% CI: 1.23–1.63), both of which were statistically significant (P < 0.001. Next, in the fully adjusted model, the HR decreased to 1.20 (95% CI: 1.04–1.39) for the TyG index and further to 1.36 (95% CI: 1.23–1.63) for TyG-AVG. However, the HR for TyG-AVG remained higher than that of the TyG index, indicating that its sensitivity and specificity in predicting delirium in patients is superior to the TyG index alone (**Tables 1**, **2**).

**Table 1:** Cox regression analysis of TyG and delirium risk in patients with acute kidney injury.

Categories	TyG model (a)		TyG model (b)		TyG model (c)	
	HR (95% CI)	*P-*value	HR (95% CI)	*P-*value	HR (95% CI)	*P*-value
**Continuous variable (per 1 unit)**	1.24 (1.10, 1.41)	<0.001	1.24 (1.09, 1.41)	<0.001	1.20 (1.04, 1.39)	0.013
Q1 (reference)	Ref		Ref		Ref	
**Q2**	1.36 (1.03, 1.80)	0.031	1.35 (1.02, 1.79)	0.037	1.06 (0.79, 1.41)	0.718
**Q3**	1.30 (0.98, 1.73)	0.070	1.29 (0.97, 1.71)	0.082	1.08 (0.80, 1.47)	0.614
**Q4**	1.74 (1.32, 2.31)	<0.001	1.72 (1.29, 2.28)	<0.001	1.43 (1.05, 1.94)	0.022

Model (a): unadjusted.

Model (b): adjusted for sex, age, and BMI.

Model (c): adjusted for multiple covariates including sepsis, atrial fibrillation, liver disease, race, platelet, PCO_2_, PO_2_, pH, potassium levels, heart rate, respiratory rate, renal disease, myocardial infarct, sex, age, BMI, CHF, SOFA, GCS, SAPS II, SIRS, APS III, RRT, peripheral vascular disease, ventilation, SBP, DBP, WBC, RBC, BUN, chronic pulmonary disease, cancer, hypertension, insulin, propofol, and diuretics.

TyG index quartiles: Q1 (6.85–8.79), Q2 (8.79–9.31), Q3 (9.31–9.83), Q4 (9.83–13.54).

TyG, triglyceride-glucose; HR, hazard ratio; CI, confidence interval; AKI, acute kidney injury.

**Table 2:** Cox regression analysis of TyG-AVG and delirium risk in the AKI population.

Categories	TyG-AVG Model (a)		TyG-AVG Model (b)		TyG-AVG Model (c)	
	HR (95% CI)	*P-*value	HR (95% CI)	*P-*value	HR (95% CI)	*P-*value
**Continuous variable (per 1 unit)**	1.41 (1.23, 1.63)	<0.001	1.41 (1.22, 1.62)	<0.001	1.36 (1.23, 1.63)	<0.001
Q1 (reference)	Ref		Ref		Ref	
**Q2**	1.20 (0.89, 1.62)	0.230	1.18 (0.87, 1.59)	0.288	1.29 (0.93, 1.78)	0.122
**Q3**	1.58 (1.17, 2.12)	0.002	1.55 (1.15, 2.09)	0.004	1.48 (1.06, 2.08)	0.021
**Q4**	1.96 (1.46, 2.64)	<0.001	1.92 (1.42, 2.59)	<0.001	1.88 (1.33, 2.65)	<0.001

Model (a): unadjusted.

Model (b): adjusted for sex, age, and BMI.

Model (c): adjusted for sepsis, atrial fibrillation, liver disease, race, platelet, PCO2, PO2, PH, potassium, heart rate, respiratory rate, renal disease, myocardial infarct, gender, age, BMI, CHF, SOFA, GCS, SAPS II, SIRS, APS III, RRT, peripheral vascular disease, ventilation, SBP, DBP, WBC, RBC, BUN, chronic pulmonary disease, cancer, hypertension, insulin, propofol, diuretics.

TyG-AVG: Q1 (7.24–8.98), Q2 (8.99–9.50), Q3 (9.50–9.98), Q4 (9.98–12.42).

TyG, triglyceride-glucose; HR, hazard ratio; CI, confidence interval; AKI, acute kidney injury.

When the TyG index and TyG-AVG were treated as categorical variables, an elevated TyG-AVG index was positively associated with an increased risk of delirium in patients with AKI in the unadjusted model. For example, in the Q3 interval, the HR was 1.58 (95% CI: 1.17–2.12), with a *P*-value of 0.002. In the Q4 interval, the HR reached 1.96 (95% CI: 1.46–2.64), with a *P*-value of < 0.001. The adjusted model yielded similar results. Among the models, individuals in the Q4 group of the TyG index in each model were significantly more likely to develop delirium in the ICU than those in other groups. This association was highly statistically significant [model (a): *P* < 0.001; model (b): *P* < 0.001; model (c): *P* = 0.022] (**Tables 1**, **2**).

**Figure 4:**
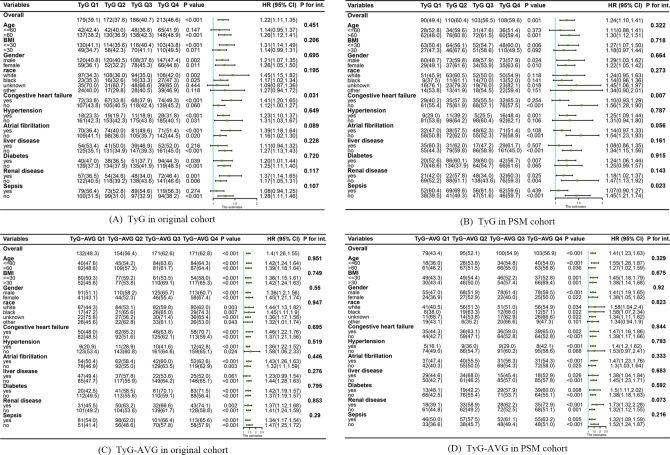
Subgroup analyses examining the relationship between triglyceride-glucose (TyG) indices and the occurrence of delirium before and after propensity score matching (PSM). **(A)** Relationship between the TyG index and the development of delirium in the original cohort. **(B)** Association between the TyG index and the occurrence of delirium occurrence in the PSM cohort. **(C)** Effect of TyG-AVG on delirium occurrence in the original cohort. **(D)** Effect of TyG-AVG on delirium occurrence in the PSM cohort.

**Figure 4** illustrates the findings from the subgroup analyses using a forest plot. Overall, the two indices exhibited consistent effects across most subgroups. However, the pre-PSM TyG index was associated with an increased likelihood of delirium in the majority of subgroups, except where influenced by factors other than CHF. In contrast, sepsis was observed in the post-PSM TyG index alongside CHF (**Figures 4A, B**). Furthermore, the TyG-AVG significantly increased the risk of delirium in the majority of subgroups and remained independent of other factors both before and after PSM (**Figures 4C, D**).

**Figure 5:**
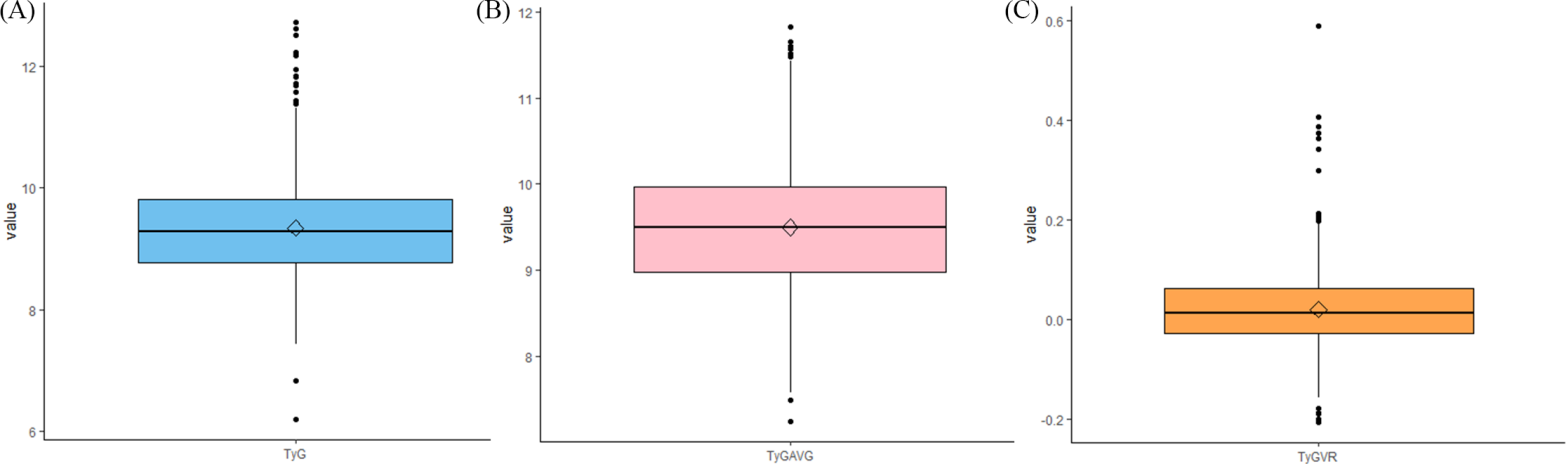
Whiskers diagrams depicting the data distributions of **(A)** TyG, **(B)** TyGAVG, and **(C)** TyGVR after PSM.

**Figure 5** illustrates the box plots for the three indices. The distribution of the data pertaining to the three indices is clearly illustrated in the graph.

## 4 Discussion

This study represents the first retrospective investigation of the linear association between TyG indices and associated outcomes. The findings indicate that patients with AKI exhibiting higher TyG-AVG values or TyG values in the ICU faced a significantly increased risk of delirium. This study offers a novel perspective on the early recognition of AKI-related delirium, suggesting that TyG indices may serve as potential biomarkers for identifying delirium in individuals with AKI. Further research is required to validate these findings.

### 4.1 Advantages of TyG indices

The TyG index in previous studies was calculated as ln(fasting triglyceride [mg/dL] × fasting glucose [mg/dL]/2) (17, 18). In other articles, such as that by Cheng et al. (2023), the time interval between the measurement of triglyceride and glucose levels used for calculating the TyG index was not explicitly defined (15). Nevertheless, in this study, a more rigorous approach to calculating the TyG index was adopted, explicitly using the triglyceride level of the patient measured upon first entry into the ICU, along with the average blood glucose levels over a 24-h period before and after the triglyceride measurement. This approach ensured that data on glucose and triglyceride levels were collected within a shorter timeframe, thereby minimising errors in the TyG calculation process and enhancing its accuracy.

TyGVR was used as an index to represent dynamic TyG (15). The study attempted to utilise TyGVR as a research metric; however, no relationship was found between delirium and this metric. A probable reason for this lack of association is that the study concluded with patient mortality, resulting in all blood glucose and triglyceride data being collected in the ICU before death. However, blood glucose and triglyceride data collected after the onset of delirium were excluded, as delirium was the primary outcome. Furthermore, the timing of delirium varied among individuals, and the proportion of data included from the ICU stay of each person was random. Therefore, the TyG average, referred to as TyG-AVG, was employed in its analysis.

Given the fluctuations in the TyG index, TyG-AVG may have produced more consistent results than the TyG index in subgroup analyses. This consistency led to a more reliable application of TyG-AVG across contexts. Additionally, a study by Cui et al. (2022) (19) highlights that the cumulative TyG index, derived from multiple years of TyG data, is more time-consuming and less convenient to use than TyG-AVG.

### 4.2 Insulin resistance, triglyceride-glucose index, acute kidney injury, and delirium risk

In some clinical studies, the TyG index has proven to be a significant biomarker for assessing IR (8, 9). The TyG index is specifically used in evaluating the biological mechanisms underlying IR, including increased mobilisation of fat in the bloodstream and abnormally elevated lipid levels (20). Compared to other methods for detecting IR, such as intravenous glucose tolerance tests, insulin suppression trials, and the homeostasis model assessment (HOMA), the TyG index offers advantages due to its broader clinical applicability. While these tests offer insights into IR status, they typically require frequent sampling and are cumbersome and complex (21). In contrast, the TyG index only requires a simple blood test, offering quicker results. This enables clinicians and researchers to access data more rapidly, facilitating timely diagnostic and treatment decisions. The TyG index only requires the measurement of triglycerides and glucose, which are typically obtained directly during routine biochemical tests in the ICU without the need for additional complex testing procedures. Consequently, the TyG index offers clinicians a quick, simple, and cost-effective tool for more accurately identifying and managing patients at risk of metabolic diseases associated with IR.

Extensive clinical research has recently focussed on the TyG index, a key metric strongly associated with various disease states. For example, Zhendong Ding et al. proposed that the TyG index was associated with complications and all-cause mortality in critically ill patients admitted to the ICU after liver transplantation. Its association with morbidity and mortality related to delirium episodes and renal disease in different populations has garnered significant attention from researchers. Sun et al. (2024), through multivariate logistic regression, demonstrated that older adults with type 2 diabetes mellitus were more susceptible to postoperative delirium. The occurrence of delirium was significantly and positively associated with a TyG index > 8.678, providing significant clinical insight into independent risk factors (12). In a study conducted by Fang et al. (2024), a significant correlation was reported between the incidence of delirium and higher TyG values (> 9.09) in patients with sepsis (13). Additionally, a multicentre survey conducted by Huang et al. (2023) indicated a significant increase in the likelihood of delirium when the TyG index exceeded 8.912 in individuals ≥ 65 years (22). This finding was consistent with the connection between the TyG index and delirium observed in the subgroup analysis in this study, specifically in patients aged > 60 years with AKI, further confirming the relevance of this index in predicting delirium risk within this specific population.

Regarding the TyG index and AKI, the study by Lv et al. (2023) revealed a significant finding: patients with AKI with a higher TyG index face a substantially increased risk of all-cause mortality (11). Further findings consistently demonstrated that the TyG index is associated with poor renal prognosis in specific patient groups. In cardiovascular disease treatment, the TyG index has been strongly associated with an increased incidence of AKI in individuals undergoing coronary revascularisation surgery and those with hypertension (23, 24). Additionally, Fang et al. (2024) investigated the TyG index in patients with sepsis and found that as the index increased, the risk of AKI in these patients increased (25). The TyG index has been independently associated with contrast-induced AKI in individuals with diabetes mellitus and acute coronary syndromes, as well as in Chinese individuals with type 2 diabetes mellitus (26, 27). These findings highlight the role of IR in AKI and suggest how risk factors in these patients should be managed in future clinical practice. In this study, the TyG index and the TyG-AVG were used, accounting for long-term trend in TyG, as biological surrogates of IR to investigate the association between patients with AKI and delirium in patients. Compared to the TyG index alone, the TyG-AVG index captures the fluctuating nature of IR, offering a more comprehensive assessment of its effect.

### 4.3 Related mechanisms

This study demonstrates a clear correlation between an increased TyG index and the risk of delirium in individuals with severe AKI. An elevated TYG index indicates abnormal insulin resistance. The cognitive impairment caused by delirium resulting from this abnormality may be associated with the following six factors:

First, glycogen synthase kinase-3 beta (GSK-3β) induces the over-phosphorylation of Tau protein, leading to cognitive impairment (28). IR in the brain increases GSK-3β activity (29). Ultimately, IR exacerbates cognitive decline. In the fructose-induced IR model, the phosphorylation of Tau protein significantly increased (30). Second, IR impairs cognitive function by increasing amyloid-beta (Aβ) protein expression (31). During the onset of IR, blood insulin levels increase. Insulin can induce the formation of Aβ oligomers in the brain (32), leading to abnormal Aβ deposition, posing a significant challenge to cellular integrity. This process causes glial cells to release numerous inflammatory factors with potent toxic effects while simultaneously releasing reactive oxygen species. Prolonged exposure to such oxidative stress may ultimately lead to cellular death (33). Third, the insulin-degrading enzyme (IDE) is crucial for identifying and degrading Aβ and insulin. When insulin levels are increased, IDE primarily degrades insulin, resulting in Aβ deposition (34). Fourth, insulin can increase the density of γ-aminobutyric acid (GABA) receptors on the postsynaptic membrane of neurons (35). GABA, an inhibitory neurotransmitter, binds more effectively to its receptors, resulting in a stronger inhibition of brain function. Fifth, in the context of IR, astrocytes can modulate the inflammatory response by secreting various cytokines, including C-reactive protein (36). When levels of these pro-inflammatory factors are significantly elevated, they may adversely affect the structure and function of the hippocampus, impairing the ability of the individual to acquire new information (37). Sixth, the frequency of the ApoE ϵ4 allele is significantly higher in patients with IR (38), and the presence of the ϵ4 is associated with decreased cognitive function (39).

Furthermore, delirium represents a deviation from baseline cognitive function (40). Several studies have shown that AKI is a significant factor in delirium in individuals (41–43). Pang et al. (2022) (44) identified six categories of clinical and pathophysiological mechanisms underlying AKI-associated delirium: impaired clearance of toxins and drugs, including sedatives; electrolyte imbalances; increased vascular permeability; fluid accumulation; depressed central dopamine turnover; and inflammation. This finding further highlights the significance of this study due to the inextricable association between AKI and delirium.

### 4.4 Study strengths and limitations

This study exhibits some strengths. First, strict control of confounding was implemented through the PSM to minimise bias. Furthermore, K–M survival, Cox regression, and subgroup analyses were used to explore and quantify the predictive efficacy of the TyG index and TyG-AVG for delirium in individuals with AKI. Additionally, the study results indicate that, compared to the existing TyGVR, the TyG-AVG exhibits a stronger early warning effect for delirium in patients with AKI, providing a more sensitive and accurate tool for clinical prognosis assessment.

This study has some limitations. First, the database did not use the gold standard, the Diagnostic and Statistical Manual of Mental Disorders, Fifth Edition (DSM-5), opting for the CAM-ICU criteria for diagnosing delirium. Consequently, the presence of hypoactive, hyperactive, or delirium phenotypes could not be predicted in this study. Secondly, This study was conducted at a single centre, with 1,378 cases of delirium observed in 2,919 individuals. To validate the reliability of these findings, a multicentre cohort network is needed to ensure the representativeness and adequacy of the data.

## 5 Conclusion

In conclusion, these findings broaden the range of existing indicators representing long-term trend in IR and reveal a significant independent association between high TyG indices and the risk of delirium in individuals with AKI in the ICU. This newly established correlation helps healthcare professionals to focus on high TyG indexes, so that individuals at higher risk of developing delirium can be identified earlier. However, since this database retrospectively does diagnose delirium using CAM-ICU, it is not possible to distinguish active, inactive, and mixed delirium in patients with AKI in actual predictions, so future studies can focus on using prospective studies and DSM-5 to diagnose delirium so that the model can predict the three types of delirium in patients with AKI, respectively, and improve the prognosis of patients with AKI.

The findings of this study align with the recent Chinese expert consensus on the management of delirium in critically ill patients, which underscores the critical importance of early detection and intervention for subclinical delirium. Utilising the TyG index to differentiate AKI patients based on their varying risks of delirium facilitates dynamic allocation of nursing resources in the ICU. Additionally, the TyG index can serve as an auxiliary reference tool for clinical decision-making. Patients should be advised to adopt a low-fat, low-sugar diet and use hypoglycemic or lipid-lowering medications to manage elevated TyG levels, thereby reducing the incidence of delirium.

## Data Availability

The original contributions presented in the study are included in the article/**Supplementary Material**, further inquiries can be directed to the corresponding author/s.
