# Supplementary material for: Associations between triglyceride-glucose indices and delirium risk in critically ill patients with acute kidney injury: a retrospective study
Source: Front Endocrinol (Lausanne). 2025 Apr 10;16:1521850. doi: 10.3389/fendo.2025.1521850 (PMC12018253; doi:10.3389/fendo.2025.1521850)
Supplement: Supplementary file 1 [file Table1.docx]

Supplementary Material

# Supplementary Tables

**Supplementary Tables 1.** Fundamental characteristics of patients with acute kidney injury.

| **Variables** | **Overall**  **(N=2919)** | **Q1**  **(N=730)** | **Q2**  **(N=731)** | **Q3**  **(N=729)** | **Q4**  **(N=729)** | **P-value** |
| --- | --- | --- | --- | --- | --- | --- |
| **Demographic** | | | | | | |
| Age, years, median [IQR] | 67.4 [56.5, 76.9] | 72.1 [60.2, 82.0] | 69.3 [58.6, 78.5] | 66.8 [56.3. 75.5] | 62.3 [52.0, 71.2] | <0.001 |
| Ethnicity, n (%) |  |  |  |  |  | 0.003 |
| White | 1682 (57.6) | 435 (59.6) | 456 (62.4) | 415 (56.9) | 376 (51.6) |  |
| Black | 366 (12.5) | 103 (14.1) | 77 (10.5) | 84 (11.5) | 102 (14.0) |  |
| Unknown | 407 (13.9) | 87 (11.9) | 92 (12.6) | 107 (14.7) | 121 (16.6) |  |
| Other | 464 (15.9) | 105 (14.4) | 106 (14.5) | 123 (16.9) | 130 (17.8) |  |
| Gender, n (%) |  |  |  |  |  | 0.283 |
| Male | 1928 (66.1) | 477 (65.3) | 474 (64.8) | 474 (65.0) | 503 (69.0) |  |
| Female | 991 (33.9) | 253 (34.7) | 257 (35.2) | 255 (35.0) | 226 (31.0) |  |
| Height, cm, median [IQR] | 170.4 [168.0, 175.0] | 170.4 [168.0, 173.0] | 170.4 [168.0, 175.0] | 170.4 [168.0, 175.0] | 170.4 [165.0, 178.0] | 0.894 |
| Weight, kg, median [IQR] | 85.5 [70.0, 100.0] | 79.2 [66.0, 93.6] | 81.6 [68.3, 95.0] | 85.5 [71.4, 100] | 93.0 [78.9, 109.0] | <0.001 |
| BMI, kg/m^2^, median [IQR] | 29.2 [24.6, 34.1] | 27.2 [23.2, 31.8] | 27.9 [23.9, 32.3] | 29.4 [24.9, 34.2] | 32.0 [27.4, 37.2] | <0.001 |
| **Vital signs,** median [IQR] | | | | | | |
| HR, min^-1^ | 87.8 [76.1, 101.1] | 85.3 [73.9, 98.1] | 88.1 [75.2, 100.2] | 87.9 [78.4, 101.0] | 90.2 [77.5, 105.1] | <0.001 |
| SBP, mmHg | 114.1 [104.7, 126.7] | 113.8 [103.6, 126.4] | 114.0 [104.2, 129.3] | 114.6 [104.9, 127.2] | 114.0 [105.7, 125.3] | 0.421 |
| DBP, mmHg | 62.0 [55.5, 69.8] | 61.6 [54.6, 69.3] | 62.4 [55.8, 70.3] | 61.7 [55.9, 70.2] | 62.2 [55.6, 69.7] | 0.306 |
| Temperature, ℃ | 36.9 [36.6, 37.2] | 36.7 [36.5, 37.0] | 36.8 [36.6, 37.1] | 36.9 [36.7, 37.3] | 37.0 [36.7, 37.4] | <0.001 |
| Respiratory rate, min^-1^ | 20.6 [17.8, 24.1] | 19.3 [17.1, 22.6] | 20.3 [17.6, 23.6] | 20.8 [18.2, 24.4] | 22.37 [19.2, 26.0] | <0.001 |
| **Laboratory tests,** median [IQR] | | | | | | |
| RBC, K/µL | 3.5 [3.0, 4.1] | 3.4 [2.9, 4.0] | 3.5 [2.9, 4.1] | 3.5 [3.0, 4.1] | 3.6 [3.1, 4.3] | 0.001 |
| PCO_2_, mmHg | 40.6 [36.9, 44.0] | 40.6 [37.0, 42.5] | 40.6 [36.3, 43.0] | 40.6 [36.7, 44.8] | 41.3 [37.0, 46.8] | <0.001 |
| PO_2_, mmHg | 108.5 [76.0, 122.2] | 110.1 [77.8, 116.1] | 110.1 [80.0, 124.2] | 108.5 [78.1, 124.8] | 100.1 [71.3, 120.1] | 0.002 |
| pH | 7.3 [7.3, 7.4] | 7.3 [7.3, 7.4] | 7.3 [7.3, 7.4] | 7.3 [7.3, 7.4] | 7.3 [7.3, 7.4] | <0.001 |
| BUN (mg/dL) | 30.3 [20.0, 48.7] | 30.5 [20.0, 48.4] | 29.0 [19.5, 46.0] | 29.0 [19.8, 45.6] | 32.5 [20.5, 53.4] | 0.025 |
| WBC, K/µL | 11.8 [8.3, 16.3] | 11.3 [8.1, 15.2] | 12.1 [8.5, 16.5] | 11.9 [8.0, 16.6] | 12.1 [8.5, 17.4] | 0.034 |
| Platelet, K/µL | 180.0 [117.0, 248.0] | 170.0 [115.5, 234.4] | 181.6 [123.7, 250.0] | 185.0 [119.1, 258.0] | 184.5 [111.5, 250.8] | 0.026 |
| Sodium, K/µL | 138.0 [135.0, 141.0] | 138.2 [135.0, 141.0] | 138.5 [135.3, 141.3] | 138.5 [135.0, 141.2] | 137.6 [134.7, 141.0] | 0.033 |
| Potassium, K/µL | 4.3 [3.9, 4.7] | 4.2 [3.8, 4.6] | 4.2 [3.8, 4.7] | 4.2 [3.8, 4.7] | 4.4 [3.9, 4.9] | <0.001 |
| **Comorbidities**, n (%) | | | | | | |
| Myocardial infarct | 677 (23.2) | 181 (24.8) | 191 (26.1) | 158 (21.7) | 147 (20.2) | 0.026 |
| Congestive heart failure | 1093 (37.4) | 324 (44.4) | 305 (41.7) | 254 (34.8) | 210 (28.8) | <0.001 |
| Peripheral vascular disease | 378 (12.9) | 97 (13.3) | 99 (13.5) | 95 (13.0) | 87 (11.9) | 0.808 |
| Hypertension | 452 (15.5) | 121 (16.6) | 123 (16.8) | 99 (13.6) | 109 (15.0) | 0.278 |
| Atrial fibrillation | 1044 (35.8) | 292 (40.0) | 283 (38.7) | 266 (36.5) | 203 (27.8) | <0.001 |
| Chronic pulmonary disease | 696 (23.8) | 183 (25.1) | 173 (23.7) | 151 (20.7) | 189 (25.9) | 0.098 |
| Liver disease | 614 (21.0) | 177 (24.2) | 156 (21.3) | 128 (17.6) | 153 (21.0) | 0.020 |
| Diabetes | 856 (29.3) | 128 (17.5) | 168 (23.0) | 247 (33.9) | 313 (42.9) | <0.001 |
| Renal disease | 861 (29.5) | 238 (32.6) | 225 (30.8) | 206 (28.3) | 192 (26.3) | 0.046 |
| Malignant cancer | 443 (15.2) | 95 (13.0) | 113 (15.5) | 126 (17.3) | 109 (15.0) | 0.155 |
| Sepsis | 1284 (44.0) | 265 (36.3) | 281 (38.4) | 324 (44.4) | 414 (56.8) | <0.001 |
| **Medications**, n (%) | | | | | | |
| Propofol | 2005 (68.7) | 429 (58.8) | 469 (64.2) | 512 (70.2) | 595 (81.6) | <0.001 |
| Insulin | 2315 (79.3) | 498 (68.2) | 545 (74.6) | 615 (84.4) | 657 (90.1) | <0.001 |
| Diuretics | 2287 (78.3) | 560 (76.7) | 587 (80.3) | 570 (78.2) | 570 (78.2) | 0.421 |
| **Severe score,** median [IQR] | | | | | | |
| SOFA | 7.0 [4.0, 10.0] | 6.0 [3.0, 9.0] | 6.0 [4.0, 9.0] | 7.0 [4.0, 10.0] | 8.0 [5.0, 11.0] | <0.001 |
| GCS | 15.0 [14.0, 15.0] | 15.0 [13.0, 15.0] | 15.0 [14.0, 15.0] | 15.0 [13.0, 15.0] | 15.0 [14.0, 15.0] | <0.001 |
| SAPS II | 43.0 [34.0, 54.0] | 42.0 [33.0, 52.0] | 43.0 [34.0, 53.0] | 43.0 [33.0, 54.0] | 45.5 [35.0, 57.0] | 0.171 |
| SIRS | 3.0 [2.0, 3.0] | 3.0 [2.0, 3.0] | 3.0 [2.0, 3.0] | 3.0 [2.0, 3.0] | 3.0 [2.0, 4.0] | <0.001 |
| APS III | 55.0 [43.0, 73.0] | 53.0 [40.0, 71.0] | 53.0 [41.0, 69.0] | 54.0 [43.0, 71.0] | 61.0 [48.0, 81.0] | <0.001 |
| **Events**, n (%) | | | | | | |
| RRT | 303 (10.4) | 56 (7.7) | 58 (7.9) | 70 (9.6) | 119 (16.3) | <0.001 |
| Ventilation | 2487 (85.2) | 577 (79.0) | 614 (84.0) | 645 (88.5) | 651 (89.3) | <0.001 |
| **Length of stay (LOS)**, median [IQR] | | | | | | |
| LOS in hospital | 16.8 [8.9, 29.0] | 14.8 [8.3, 26.0] | 17.7 [9.2, 29.1] | 17.3 [8.9, 29.9] | 17.8 [9.3, 30.1] | 0.008 |
| LOS in ICU | 6.5 [2.8, 13.6] | 5.0 [2.3, 10.2] | 5.8 [2.8, 12.3] | 6.3 [2.6, 14.1] | 9.9 [3.8, 18.4] | <0.001 |
| **Outcome,** n (%) | | | | | | |
| Delirium | 1378 (47.2) | 307 (42.1) | 321 (43.9) | 318 (43.6) | 432 (59.3) | <0.001 |

Abbreviations: TyG index, triglyceride-glucose index; BMI, body mass index; HR, heart rate; SBP, systolic blood pressure; DBP, diastolic blood pressure; RBC, red blood cell; BUN, blood urea nitrogen; WBC, white blood cell; SOFA, sequential organ failure assessment; SAPS II, simplified acute physiological score II; GCS, Glasgow Coma Scale; SIRS, systemic inflammatory response syndrome; APS III, acute physiology score III; RRT, renal replacement therapy; ICU, intensive care unit; IQR, interquartile range.

a. TyG index: Q1 (6.85–8.79), Q2 (8.79–9.31), Q3 (9.31–9.83), and Q4 (9.83–13.54).

b. Delirium was defined as a positive result obtained through systematic assessment using a standardised tool, the CAM.

**Supplementary Tables 2.** Baseline characteristics before and after propensity score matching in both groups.

| **Variables** | **Original Cohort** | | **P-value** | **Matched Cohort** | | **P-value** |
| --- | --- | --- | --- | --- | --- | --- |
|  | **TyG detected once during ICU stay (N=1828)** | **TyG detected ≥ 2 times during ICU stay (N=1091)** |  | **TyG detected once during ICU stay (N=727)** | **TyG detected ≥ 2 times during ICU stay (N=727)** |  |
| TyG index, median [IQR] | 9.1 [8.6, 9.6] | 9.5 [9.0, 10.2] | <0.001 | 9.3 [8.8, 9.8] | 9.3 [8.8, 9.8] | 0.331 |
| TyG-AVG, median [IQR] | NA | 9.7 [9.1, 10.2] | NA | NA | 9.5 [9.0, 10.0] | NA |
| **Demographic** | | | | | | |
| Age, years, median [IQR] | 69.8 [58.8, 79.5] | 64.0 [52.2, 72.7] | <0.001 | 66.5 [56.3, 75.5] | 66.0 [55.6, 75.0] | 0.793 |
| Sex, n (%) |  |  | 0.070 |  |  | 0.366 |
| Male | 1185 (64.8) | 743 (68.1) |  | 490 (67.4) | 506 (69.6) |  |
| Female | 643 (35.2) | 348 (31.9) |  | 237 (32.6) | 221 (30.4) |  |
| Ethnicity, n (%) |  |  | <0.001 |  |  | 0.596 |
| White | 1107 (60.6) | 575 (52.7) |  | 410 (56.4) | 412 (56.7) |  |
| Black | 219 (12.0) | 147 (13.5) |  | 87 (12.0) | 97 (13.3) |  |
| Unknown | 233 (12.7) | 174 (15.9) |  | 103 (14.2) | 108 (14.9) |  |
| Other | 269 (14.7) | 195 (17.9) |  | 127 (17.5) | 110 (15.1) |  |
| Height, cm, median [IQR] | 170.4 [168.0, 175.0] | 170.4 [165.0, 175.0] | <0.001 | 170.4 [165.0, 177.8] | 170.4 [168.0, 175.0] | 0.115 |
| Weight, kg, median [IQR] | 82.7 [68.5, 97.7] | 89.7 [75.0, 104.3] | <0.001 | 85.5 [71.0, 101.7] | 87.0 [72.3, 100.7] | 0.970 |
| BMI, kg/m^2^, median [IQR] | 28.1 [24.0, 33.1] | 30.8 [26.0, 35.6] | <0.001 | 29.4 [25.0, 34.8] | 30.1 [25.1, 34.2] | 0.986 |
| **Vital signs**, median [IQR] | | | | | | |
| HR, min^-1^ | 86.6 [75.4, 98.8] | 90.0 [78.2, 104.3] | <0.001 | 88.4 [77.1, 102.3] | 88.8 [76.5, 103.1] | 0.809 |
| SBP, mmHg | 115.0 [104.7, 129.8] | 112.5 [104.8, 123.2] | <0.001 | 112.3 [104.3, 123.8] | 112.9 [104.8, 124.3] | 0.851 |
| DBP, mmHg | 62.0 [55.2, 70.5] | 62.1 [56.0, 68.5] | 0.741 | 60.7 [54.1, 68.1] | 61.7 [55.4, 68.8] | 0.023 |
| Respiratory rate, min^-1^ | 20.0 [17.5, 23.2] | 22.0 [18.6, 25.6] | <0.001 | 20.9 [18.2, 24.4] | 21.0 [18.1, 24.3] | 0.648 |
| Temperature, ℃ | 36.8 [36.6, 37.1] | 37.0 [36.6, 37.3] | <0.001 | 36.9 [36.6, 37.3] | 36.9 [36.6, 37.2] | 0.747 |
| **Laboratory tests**, median [IQR] | | | | | | |
| RBC, K/µL | 3.5 [3.0, 4.1] | 3.5 [2.9, 4.1] | 0.082 | 3.4 [3.0, 4.0] | 3.4 [2.9, 4.0] | 0.361 |
| PCO_2_, mmHg | 40.6 [37.0, 42.7] | 42.4 [36.7, 46.3] | <0.001 | 40.6 [37.0, 45.3] | 41.2 [36.0, 44.3] | 0.656 |
| PO_2_, mmHg | 110.1 [78.3, 119.2] | 101.6 [74.8, 124.8] | 0.001 | 110.0 [77.2, 130.3] | 106.0 [75.0, 129.1] | 0.129 |
| pH | 7.3 [7.3, 7.4] | 7.3 [7.3, 7.4] | <0.001 | 7.3 [7.3, 7.4] | 7.3 [7.3, 7.4] | 0.461 |
| BUN (mg/dL) | 30.7 [20.0, 49.0] | 30.0 [19.0, 48.3] | 0.167 | 30.0 [20.0, 48.7] | 30.0 [20.0, 49.2] | 0.858 |
| WBC, K/µL | 11.6 [8.3, 15.7] | 12.2 [8.2, 17.5] | 0.086 | 12.3 [8.5, 17.0] | 12.1 [8.2, 17.6] | 0.730 |
| Platelet, K/µL | 180.5 [122.0, 248.0] | 176.8 [110.0, 248.0] | 0.156 | 178.0 [106.0, 243.7] | 172.0 [107.5, 240.0] | 0.550 |
| Sodium, K/µL | 138.5 [135.2, 141.0] | 138.0 [134.6, 141.0] | 0.005 | 138.3 [135.0, 141.3] | 138.0 [134.7, 141.3] | 0.384 |
| Potassium, K/µL | 4.2 [3.8, 4.7] | 4.3 [3.9, 4.8] | <0.001 | 4.2 [3.9, 4.8] | 4.3 [3.9, 4.7] | 0.582 |
| **Comorbidities**, n (%) | | | | | | |
| Myocardial infarct | 491 (26.9) | 186 (17.0) | <0.001 | 139 (19.1) | 151 (20.8) | 0.431 |
| Congestive heart failure | 740 (40.5) | 353 (32.4) | <0.001 | 254 (34.9) | 257 (35.4) | 0.869 |
| Peripheral vascular disease | 250 (13.7) | 128 (11.7) | 0.130 | 92 (12.7) | 90 (12.4) | 0.874 |
| Hypertension | 319 (17.5) | 133 (12.2) | <0.001 | 112 (15.4) | 106 (14.6) | 0.659 |
| Atrial fibrillation | 678 (37.1) | 366 (33.5) | 0.053 | 265 (36.5) | 262 (36.0) | 0.870 |
| Chronic pulmonary disease | 437 (23.9) | 259 (23.7) | 0.919 | 189 (26.0) | 164 (22.6) | 0.126 |
| Liver disease | 366 (20.0) | 248 (22.7) | 0.082 | 191 (26.3) | 182 (25.0) | 0.589 |
| Diabetes | 536 (29.3) | 320 (29.3) | 0.996 | 216 (29.7) | 106 (14.6) | 0.089 |
| Renal disease | 608 (33.3) | 253 (23.2) | <0.001 | 187 (25.7) | 195 (26.8) | 0.634 |
| Malignant cancer | 251 (13.7) | 192 (17.6) | 0.005 | 134 (18.4) | 132 (18.2) | 0.892 |
| Sepsis | 652 (35.7) | 632 (57.9) | <0.001 | 380 (52.3) | 363 (49.9) | 0.372 |
| **Medications**, n (%) | | | | | | |
| Propofol | 1058 (57.9) | 947 (86.8) | <0.001 | 619 (85.1) | 594 (81.7) | 0.078 |
| Insulin | 1391 (76.1) | 924 (84.7) | <0.001 | 607 (83.5) | 586 (80.6) | 0.151 |
| Diuretics | 1357 (74.2) | 930 (85.2) | <0.001 | 607 (83.5) | 594 (81.7) | 0.369 |
| **Severe score**, median [IQR] | | | | | | |
| SOFA | 6 [4, 9] | 8.0 [5.0, 11.0] | <0.001 | 7.0 [5.0, 11.0] | 7.0 [5.0, 11.0] | 0.579 |
| SAPS II | 42 [33, 52] | 45.0 [35.0, 56.0] | <0.001 | 45.0 [36.0, 56.0] | 45.0 [36.0, 56.0] | 0.958 |
| GCS | 15 [13, 15] | 15.0 [14.0, 15.0] | 0.023 | 15.0 [14.0, 15.0] | 15.0 [14.0, 15.0] | 0.775 |
| SIRS | 3.0 [2.0, 3.0] | 3.0 [2.0, 4.0] | <0.001 | 3.0 [2.0, 4.0] | 3.0 [2.0, 4.0] | 0.866 |
| APS III | 52.0 [41.0, 69.0] | 59.0 [47.0, 79.0] | <0.001 | 59.0 [45.0, 77.0] | 57.0 [45.0, 76.0] | 0.675 |
| **Events**, n (%) | | | | | | |
| RRT | 147 (8.0) | 156 (14.3) | <0.001 | 93 (12.8) | 91 (12.5) | 0.875 |
| Ventilation | 1499 (82.0) | 988 (90.6) | <0.001 | 661 (90.9) | 646 (88.9) | 0.192 |
| **Length of stay (LOS)** | | | | | | |
| LOS in hospital | 13.4 [7.2, 22.6] | 24.9 [14.6, 39.8] | <0.001 | 19.4 [10.1, 30.3] | 20.2 [12.0, 31.7] | 0.146 |
| LOS in ICU | 4.8 [2.3, 9.8] | 11.6 [5.1, 20.8] | <0.001 | 8.2 [4.0, 15.1] | 8.4 [3.4, 14.5] | 0.433 |
| **Outcome**, n (%) | | | | | | |
| Delirium | 750 (41.0) | 628 (57.6) | <0.001 | 411 (56.5) | 377 (51.9) | 0.074 |

Abbreviations: PSM, propensity score matching; TyG index, triglyceride-glucose index; TyG-AVG, triglyceride-glucose average; NA, not available; BMI, body mass index; HR, heart rate; SBP, systolic blood pressure; DBP, diastolic blood pressure; RBC, red blood cell; BUN, blood urea nitrogen; WBC, white blood cell; SOFA, sequential organ failure assessment; SAPS II, simplified acute physiological score II; GCS, Glasgow Coma Scale; SIRS, systemic inflammatory response syndrome; APS III, acute physiology score III; RRT, renal replacement therapy; ICU, intensive care unit; IQR, interquartile range.
